# Control of stilbene conformation and fluorescence in self-assembled capsules

**DOI:** 10.3762/bjoc.5.79

**Published:** 2009-12-11

**Authors:** Mark R Ams, Dariush Ajami, Stephen L Craig, Jye-Shane Yang, Julius Rebek

**Affiliations:** 1 The Skaggs Institute for Chemical Biology and Department of Chemistry, The Scripps Research Institute, 10550 North Torrey Pines Road, La Jolla, CA 92037, U.S., Tel: 858 784 2250; Fax: 858 784 2876; 2 Department of Chemistry, Duke University, Durham, NC, 27708-0346, U.S.; 3 Department of Chemistry, National Taiwan University, No. 1. Sec. 4, Roosevelt Road, Taipei, 10617, Taiwan

**Keywords:** molecular twisting, quenching, reversible encapsulation, self-assembly, stilbene fluorescence

## Abstract

The extensively studied *trans*-stilbene molecule is known to give only weak fluorescence in solution and inside loosely-fitting synthetic capsules. However, *trans*-stilbene has been recently studied in the context of antibody interiors, where binding results in strong blue fluorescence. The present research was undertaken to understand the spatial factors that influence stilbene fluorescence. *trans*-Stilbene was encapsulated in the snug, self-assembled complex **1.1** and exhibited fluorescence quenching due to the distortion of its ground-state geometry. When the complex is elongated by incorporating glycouril spacers, *trans*-stilbene is allowed to adapt a fully coplanar arrangement and fluorescence returns.

## Introduction

**Figure 1:**
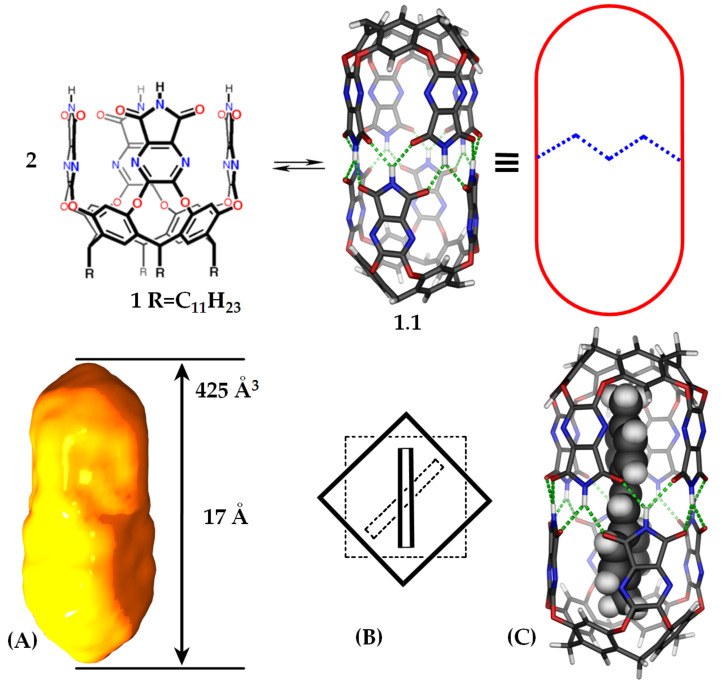
(Top) Tetraimide cavitand **1**, the dimeric capsule **1.1** and its cartoon representation. (Bottom) The shape of the space inside (A), a schematic view along the central axis with two aromatic guests (B) and an energy-minimized (AM1) complex of stilbene in **1.1** (C).

The fluorescence of *trans*-stilbene has been extensively researched [1], and weak fluorescence occurs in aqueous solutions or typical organic solvents. In a highly structured environment such as an antibody interior [2–4], recent studies show that nearby tryptophans can transfer electrons to the stilbene excited state and an intense blue fluorescence develops. Inside the tight-fitting capsule **1.1** [5–6] (Figure 1) where it is surrounded by 16 aromatic panels, *trans*-stilbene’s fluorescence is reduced to only 2% of what is observed in bulk solution. In contrast, normal fluorescence is observed in a loose-fitting capsule [7] although the photostationary *trans*-/*cis*-isomerization equilibria are altered in the limited space [8]. Isomerization of *trans*- to *cis*-stilbene is not possible in **1.1** but little else is known about the photophysics of guests in this capsule. This research was undertaken to understand what controls the behavior of stilbenes in this and related constrained environments.

## Results and Discussion

The space inside capsule **1.1** is defined by two pyramids comprising resorcinarenes at the ends and square prisms of the heterocyclic walls near the middle. The quenching of stilbene fluorescence may be an effect of the fixed aromatics of the resorcinarene or the heterocyclic walls, but we surmised that there was a subtler cause. As seen in the skeletal model **1.1** of the space within depicted in Figure 1A, the square prisms are twisted by about 45° along their long axes. A typical aromatic guest such as benzene fits best when it is nestled diagonally in a prism’s space. Accordingly, the aromatic rings of longer molecules such as biphenyls and stilbenes cannot be coplanar in their lowest energy conformations inside **1.1.** Rather, they must be twisted by 45° or so along their rotatable internal bonds.

**Figure 2:**
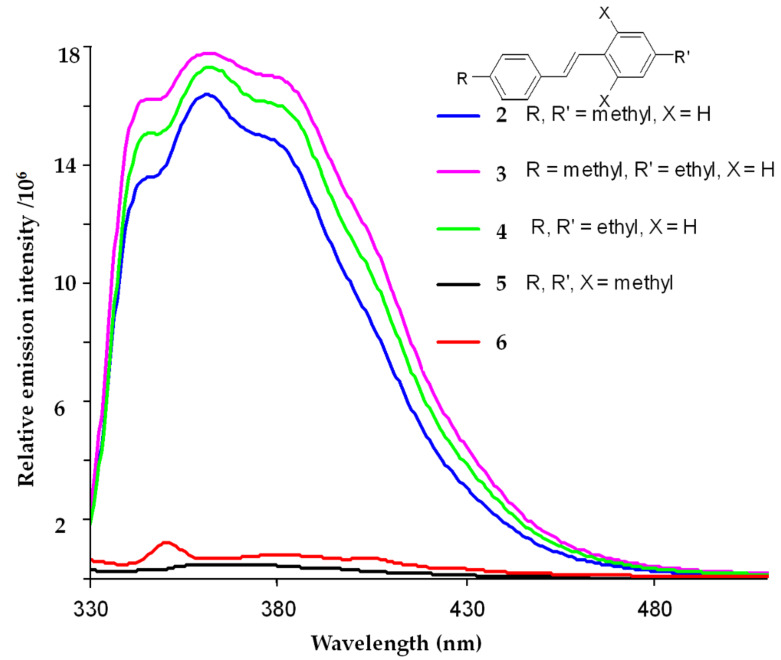
Room temperature fluorescence spectra at λ_exc_ = 318 nm for 10 µM mesitylene solutions of 4,4′-dimethylstilbene (**2**); 4-ethyl-4′-methylstilbene (**3**); 4,4′-diethylstilbene (**4**); 2,4,4′,6-tetramethylstilbene (**5**) and assembly **6** (λ_exc_ = 300 nm).

Among guests of **1.1**, 4,4′-dimethylstilbene (**2**) provides an excellent fit; it fills about 53% of the capsule’s space and the methyl groups of the guest can access the tapered ends of the host. The homologue, 4-ethyl-4′-methylstilbene (**3**), is also encapsulated (see Supporting Information File 1 for NMR spectrum), but the slightly longer 4,4′-diethyl derivative **4** simply does not fit. Figure 2 illustrates the effect of encapsulation on **3** (assembly **6**). The fluorescence is 96% quenched when λ_exc_ = 318 nm. For comparison, the emission of the permanently twisted, *o*-substituted stilbene **5** (λ_exc_ = 300 nm) is also shown [9].

**Figure 3:**
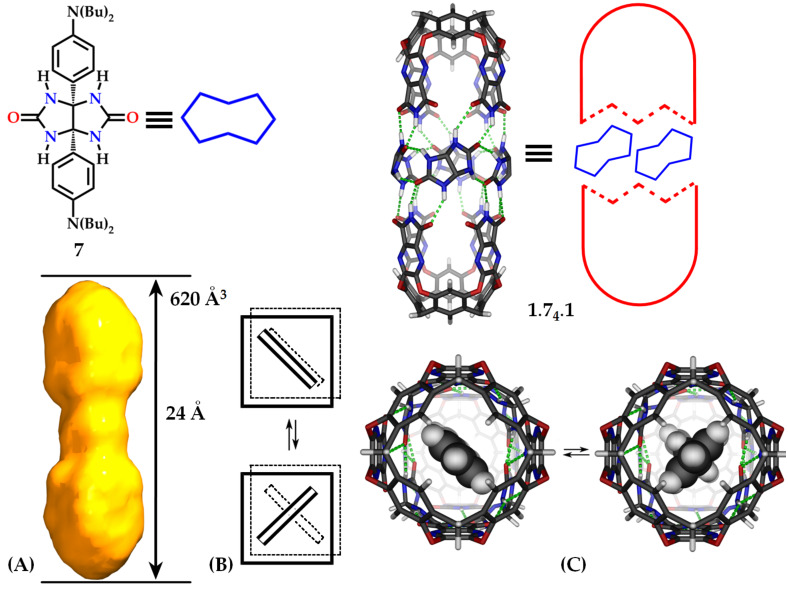
(Top) Glycouril **7**, the extended capsule **1.7_4_.1**, (only one enantiomeric arrangement is shown) and its cartoon representation. The shape of the space inside (A) a schematic view along the central axis with two aromatic guests (B) and an end-on view of an energy-minimized complex of stilbene inside (C).

**Figure 4:**
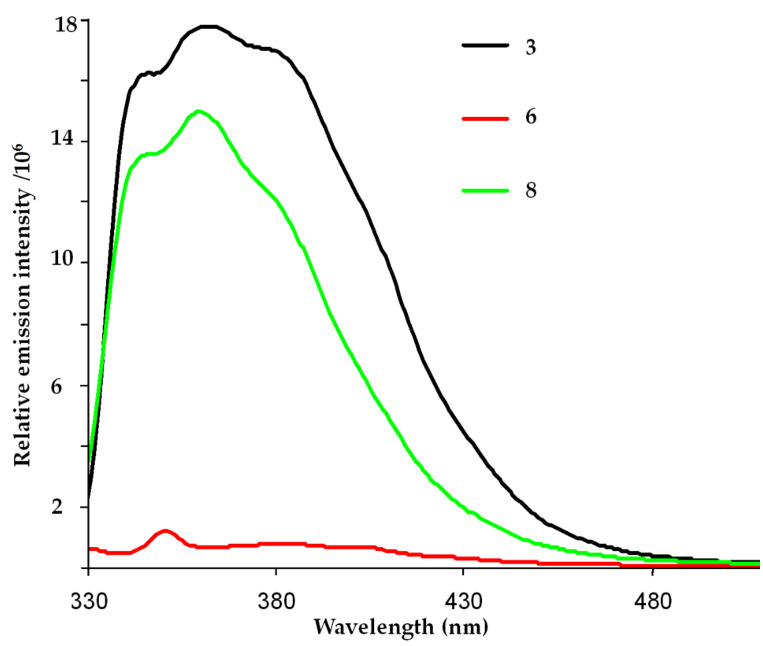
Room temperature emission spectra for 10 µM solutions of 4-ethyl-4′-ethylstilbene (**3**) in the capsule **1.1** (**6**) and **1.7_4_.1** (**8**). λ_exc_ = 318 nm.

Can the fluorescence of the encapsulated stilbene be restored? When suitable guests are present, addition of glycolurils such as **7** to solutions of **1.1** generates extended capsule **1.7_4_.1** (Figure 3) [10]. The glycolurils are arranged in a chiral manner, in either cycloenantiomer of the extended capsule. The glycolurils force the two square prisms of **1.7_4_.1** into registry (Figure 3A). That is, the square prisms are now aligned and stilbene as well as related guests may be found either in the fully coplanar arrangement favored by extended resonance stabilization (Figure 3B) or at another minimum with a 90° dihedral angle between their aromatic rings. 4-Ethyl-4′-methylstilbene (**3**) is a guest for **1.7_4_.1** when CD_2_Cl_2_ is a co-guest (see Supporting Information File 1 for NMR spectrum). In the fluorescence experiments at λ_exc_ = 318 nm, the fluorescence of 4-ethyl-4′-methylstilbene **3** and assembly **8** is restored, as shown in Figure 4. As a control experiment to test for the possibility of any fluorescence contribution from assembly **1.7_4_.1**, the fluorescence of the complex of **1.7_4_.1** with alkyl chain C_17_H_36_ was also investigated. No additional fluorescence was observed when C_17_H_36_ was a guest (see Supporting Information File 1 for fluorescence and NMR spectra).

Earlier we showed that it is possible to reversibly interconvert capsules **1.1** and **1.7_4_.1**. The weakly basic glycoluril spacer is protonated by addition of gaseous HCl and precipitates in typical organic solutions. The remaining components reassemble to the original capsule **1.1** [11]. Subsequent addition of NEt_3_ releases the glycoluril into solution and restores the extended capsule **1.7_4_.1**. This was shown previously to be a fully reversible process with long chain alkane guests. We are currently pursuing this application with stilbenes and studying the exchange of subunits in the process [12–13].

Many fluorescent sensors have been reported in the literature [14–17], however they usually respond to chemical changes rather than purely geometrical ones. Here, self-assembly of an external host system is responsible for turning on and off stilbene fluorescence through geometrical control of the stilbene’s surroundings.

## Supporting Information

File 1 Control of stilbene conformation and fluorescence in self-assembled capsules.
