# Supplementary material for: Control of stilbene conformation and fluorescence in self-assembled capsules
Source: Beilstein J Org Chem. 2009 Dec 11;5:79. doi: 10.3762/bjoc.5.79 (PMC2839510; doi:10.3762/bjoc.5.79)
Supplement: File 1 — Control of stilbene conformation and fluorescence in self-assembled capsules. [file Beilstein_J_Org_Chem-05-79-s001.pdf]

Control of stilbene conformation and fluorescence in self-assembled capsules

Supporting Information

Mark R. Ams [1], Dariush Ajami[1], Stephen L. Craig[2], Jye-Shane Yang[3] and Julius Rebek, Jr.[*1]

Address: [1]The Skaggs Institute for Chemical Biology and Department of Chemistry

The Scripps Research Institute, 10550 North Torrey Pines Road, La Jolla, CA 92037, U.S., Tel: 858.784.2250; Fax: 858.784.2876, [2]Department of Chemistry, Duke University, Durham, NC, 27708-0346, U.S. and [3]Department of Chemistry, National Taiwan University, No. 1. Sec. 4, Roosevelt Road, Taipei, 10617, Taiwan

Email: Julius Rebek, Jr. - jrebek@scripps.edu

* Corresponding author

General Experimental

NMR spectra were recorded on a Bruker DRX-600 spectrometer with a 5 mm QNP probe. The chemical shifts are given in parts per million (ppm) on the delta scale (δ), and the coupling constant values (*J*) are in Hertz. The solvent peak was used as the reference value. Deuterated solvents were obtained from Cambridge Isotope Laboratories, Inc., Andover, MA, and used without further purification. For ^{1}H NMR: $CDCl_3$ = 7.27 ppm; 1,3,5-trimethylbenzene-d_{12}. For ^{13}C NMR: $CDCl_3$ = 77.23 ppm; 1,3,5-trimethylbenzene-d_{12}. Abbreviations for NMR data: s = singlet; d = doublet; t = triplet; q = quartet; dd = doublet of doublets; dt = doublet of triplets; dq = doublet of quartets; tt = triplet of triplets; m = multiplet; br = broad; app = apparent, ABq = AB quartet.

High resolution mass spectra (HRMS) were recorded on an Agilent ESITOF mass spectrometer by Scripps Center for Mass Spectrometry. UV absorption spectra were recorded on a Varian Cary 50 UV-Visible spectrophotometer. Fluorescence measurements were obtained using a Fluorolog-3 Model FL3-

21 spectrofluorometer. Mesitylene used for the spectroscopy studies was distilled to remove impurities beforehand.

Thin layer chromatography (TLC) was performed using E. Merck silica gel 60F-254 (0.25 mm) analytical glass plates. Light absorption by compounds was observed using ultraviolet light (254 nm or 365 nm). Silica gel columns for flash chromatography, according to the method of Still, were prepared with Silicycle silica gel 60 Å (230–240 mesh).

Experimental Procedures

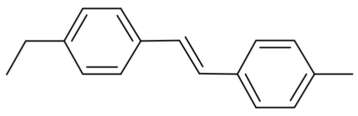

3. To a solution of 4-methylbenzyl bromide (10.0 g, 40 mmol) in THF was added triphenylphosphine (13.6 g, 52 mmol). The solution was heated at reflux for 8 h. The resulting solid was filtered and washed with hexanes (2 × 50 mL) and ether (2 × 50 mL) to afford (4-methylbenzyl)triphenylphosphonium bromide [1]. This compound was used without further purification.

To a solution of (4-methylbenzyl)triphenylphosphonium bromide (400 mg, 0.89 mmol), 4-ethylbenzaldehyde (0.13 mL, 0.982 mmol) in CH_2Cl_2 (50 mL) was added freshly powdered potassium hydroxide (110 mg, 1.97 mmol) and the reaction was stirred at room temperature for 2 h. Volatiles from the reaction mixture were removed *in vacuo* and the remaining solid was recrystallized from EtOH (20 mL) to afford **3** as a white crystalline solid (50 mg, 25%): ^{1}H NMR (600 MHz, $CDCl_3$) δ 7.46–7.42 (m, 4 H), 7.22–7.07 (m, 4 H), 2.70–2.65 (q, 2 H), 2.39 (s, 3 H), 1.29–1.26 (t, 3 H); ^{13}C NMR (150 MHz, $CDCl_3$) δ 143.89, 137.46, 135.21, 134.97, 129.56, 128.37, 127.91, 127.86, 126.59, 126.52, 28.85, 21.45, 15.76; HRMS *m/e* calcd for $C_{17}H_{18}$ 222.32, found 222.14084.

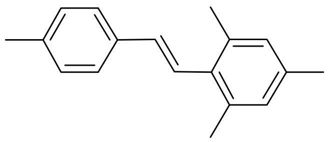

5. [2] To a solution of (4-methylbenzyl)triphenylphosphonium bromide (3.3 g, 7.43 mmol), mesitaldehyde (0.98 mL, 6.757 mmol) in CH_2Cl_2 (13 mL) was added freshly powdered potassium hydroxide (834 mg, 14.86 mmol) and the reaction was stirred at room temperature for 6 h. Volatiles from the reaction mixture were removed *in vacuo* and the remaining solid was purified by column chromatography (SiO_2, hexanes) to afford **5** as a white crystalline solid (70 mg, 4%): ^1H NMR (600 MHz, $CDCl_3$) δ 7.46–7.44 (d, *J* = 12 Hz, 2 H), 7.27–7.22 (d, *J* = 12 Hz, 2 H), 7.12–7.09 (d, *J* = 16 Hz, 1 H), 6.96 (s, 2 H), 6.63–6.60 (d, *J* = 16 Hz, 1 H), 2.42 (s, 3 H), 2.40 (s, 6 H), 2.35 (s, 3 H); ^{13}C NMR (150 MHz, $CDCl_3$) δ , 137.4, 136.3, 135.1, 134.3, 133.7, 129.5, 128.8, 126.3, 126.1; HRMS *m/e* calcd for $C_{18}H_{20}$ 236.35, found 236.1564.

Absorption Spectra (distilled mesitylene, 10 μM)

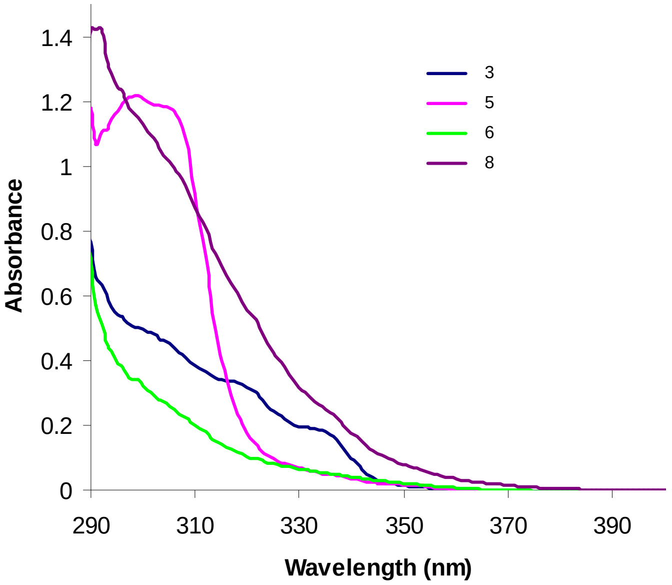

Fluorescence Spectra (distilled mesitylene, 10 μM, λ_{exc} = 318 nm)

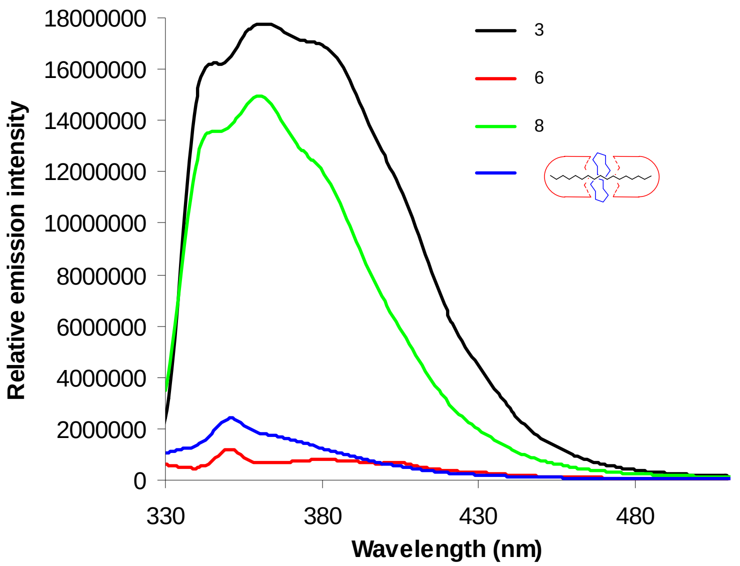

Preparation of Spectroscopy Solutions

Assembly 6:

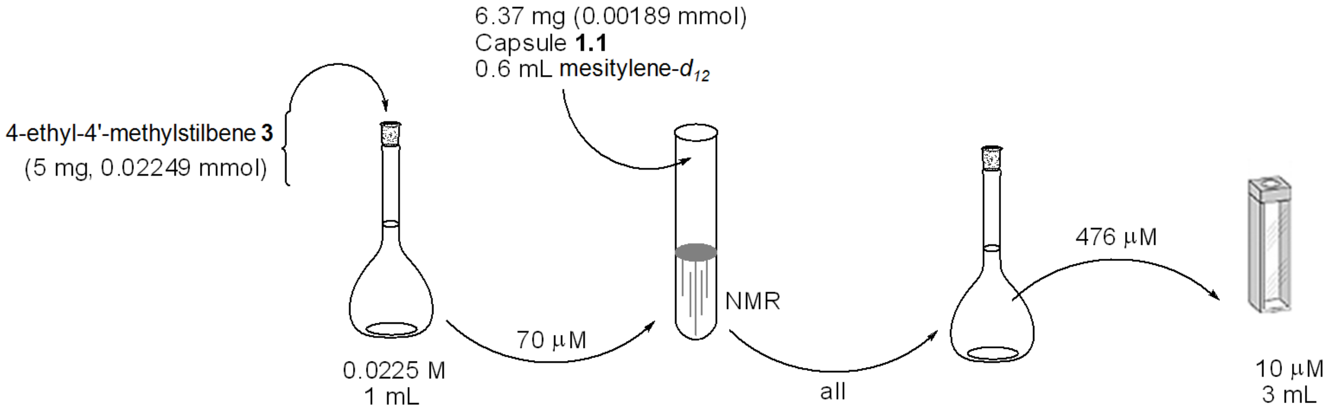

Assembly 8:

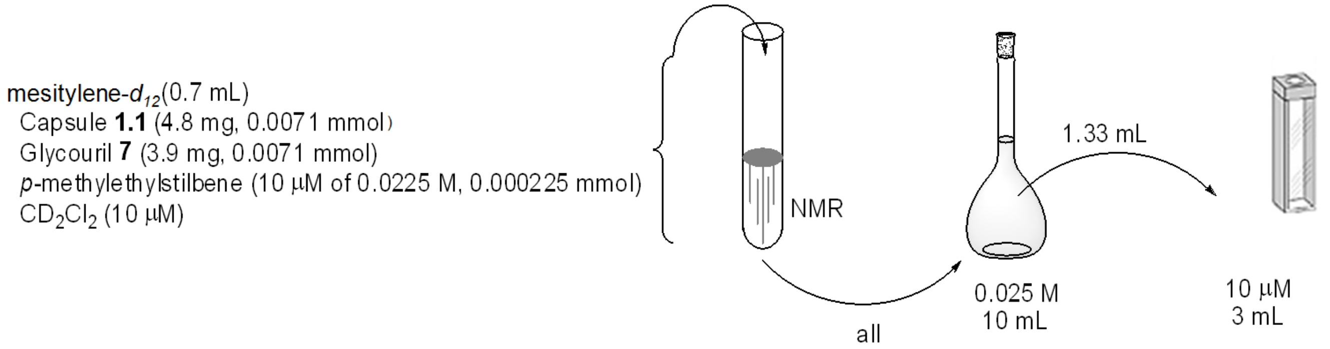

NMR Spectra

Compound **3** ($CDCl_3$). ^1H NMR.

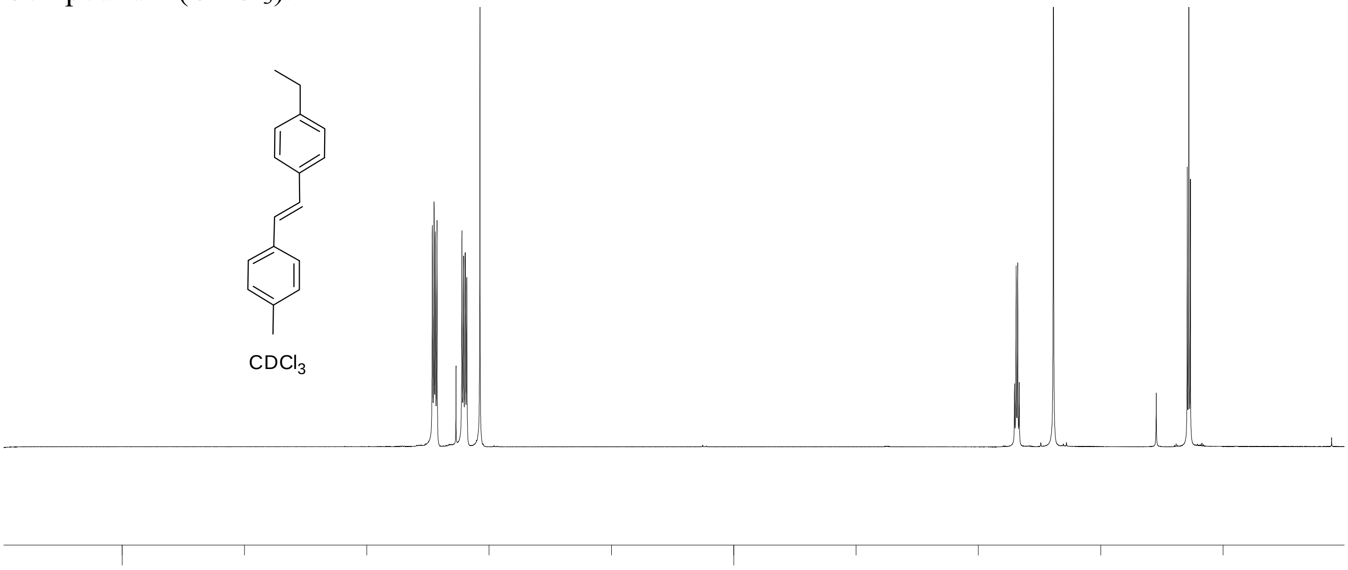

Compound **3** ($CDCl_3$). ^{13}C NMR.

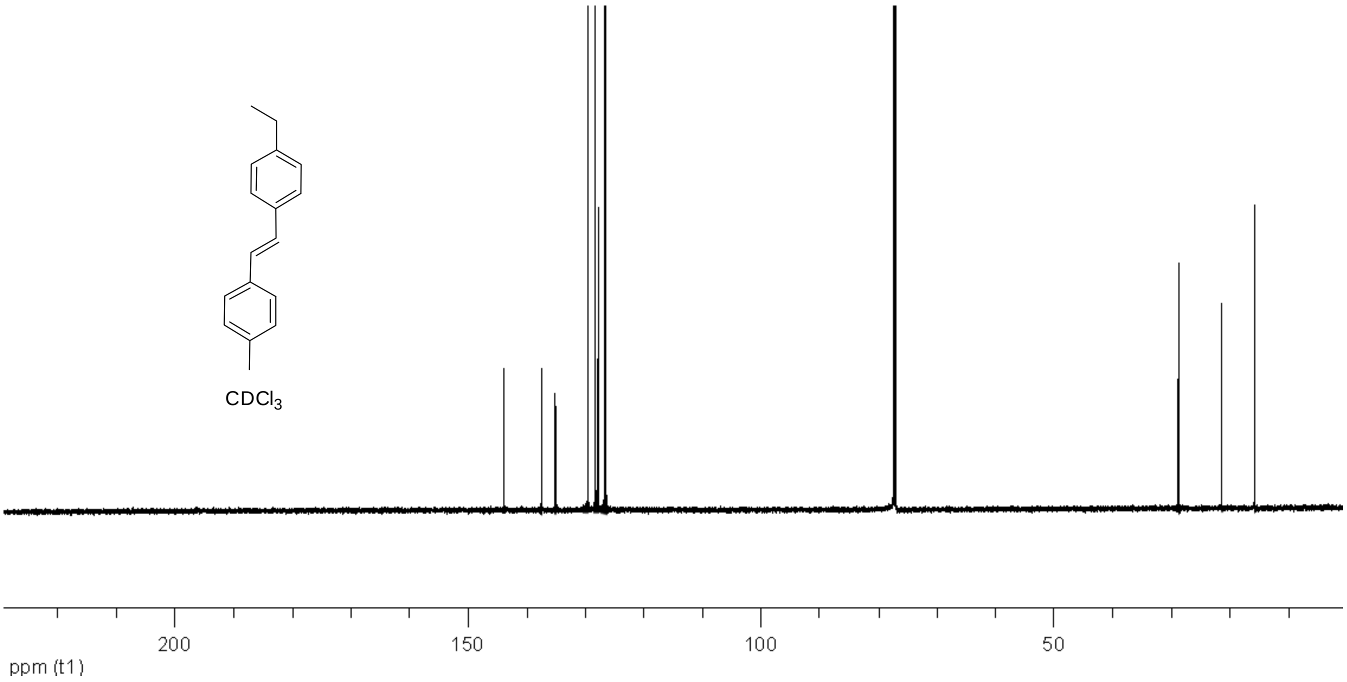

Compound **5** ($CDCl_3$). ^{1}H NMR.

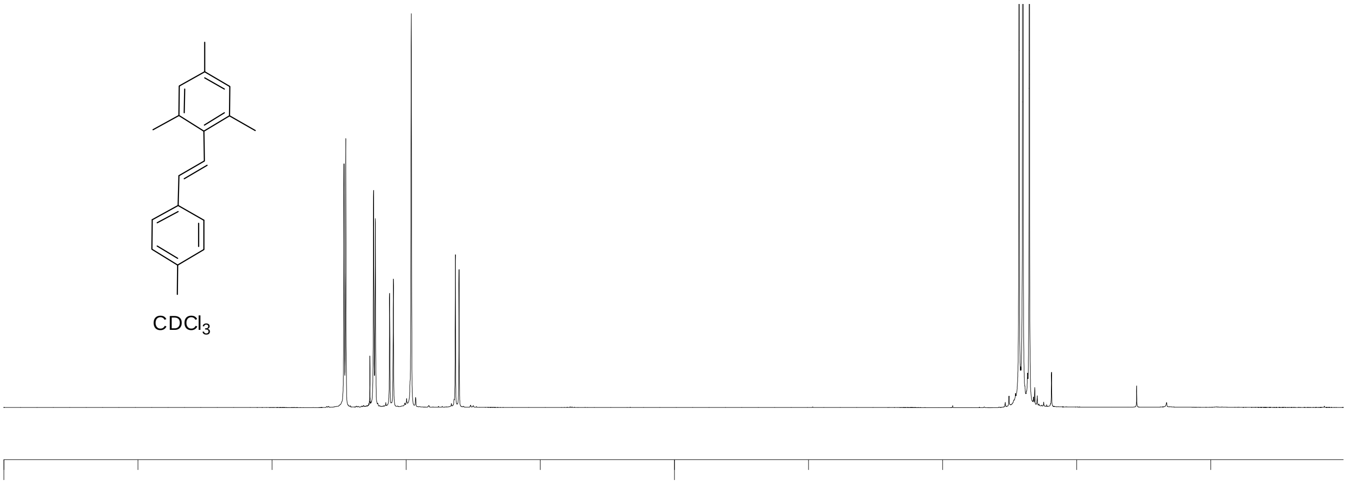

Compound **5** ($CDCl_3$). ^{13}C NMR.

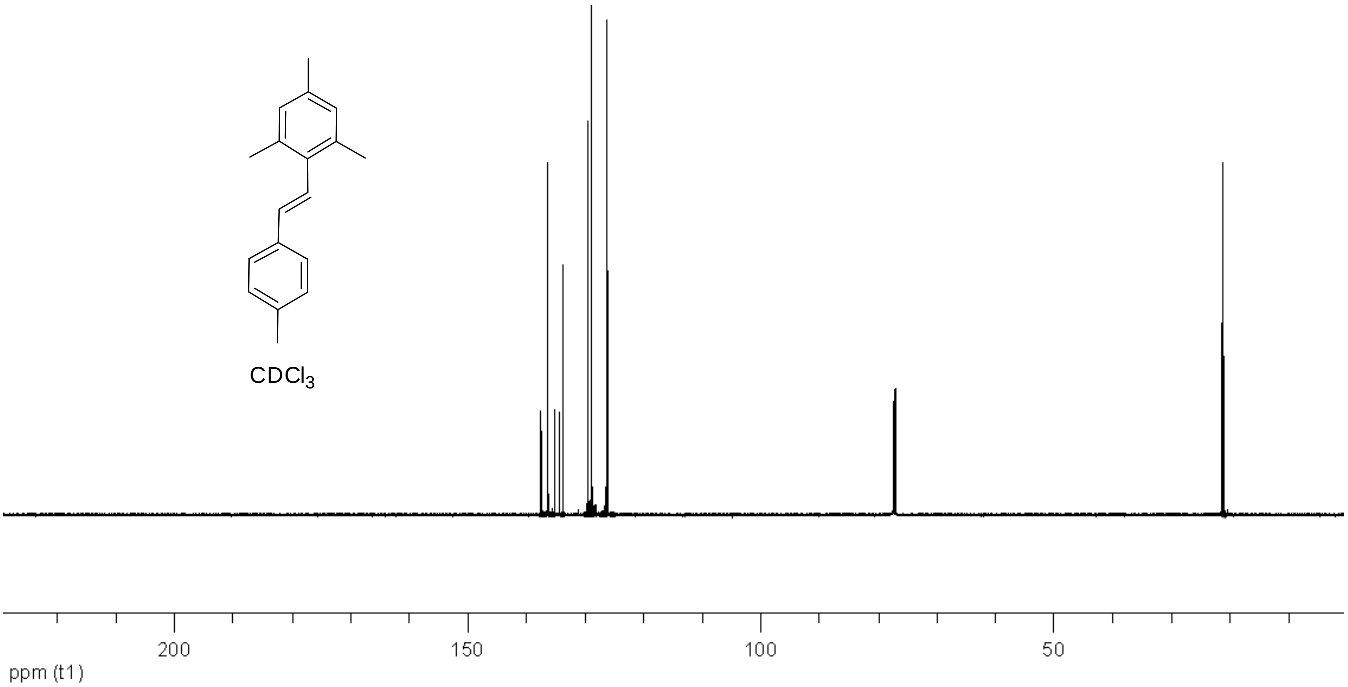

Formation of the guest-host complexes **6** and **8** in mesitylene-d_{12} was established through ^{1}H NMR experiments at millimolar concentrations. These solutions are then diluted to 10 μM for the fluorescence experiments. In earlier reports we showed using FRET that assemblies of **1.1** can form at concentrations

as low as 250 nM [3]. For the extended capsule **1.8₄.1** containing $C_{17}H_{36}$ guest, we confirmed this assembly at 10 μM using ^{1}H NMR spectroscopy. The assembly remains stable after two weeks.

Compound **8** (mesitylene-d_{12}). ^{1}H NMR. 321 μM

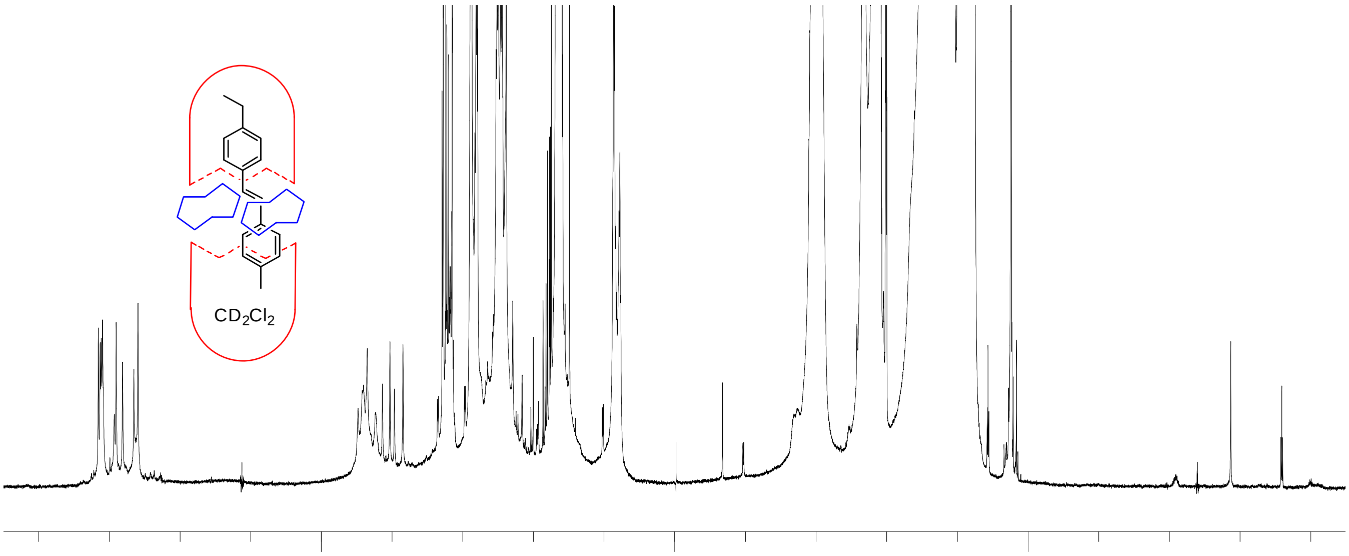

$C_{17}H_{36}$ inside **1.7₄.1** (mesitylene-d_{12}). ^{1}H NMR. 2.375 mM

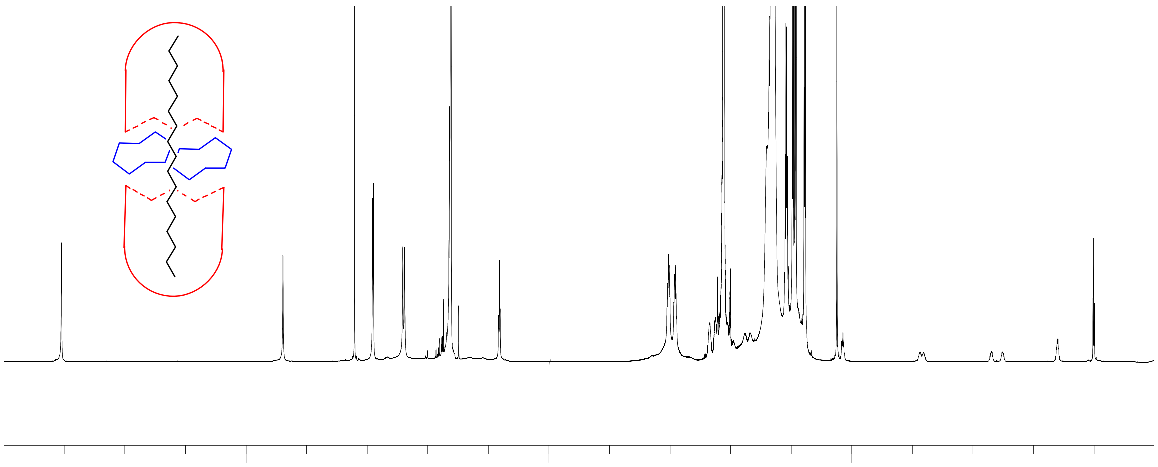

$C_{17}H_{36}$ inside **1.7$_4$.1** (mesitylene-d_{12}). ^{1}H NMR. 10 µM. Contains characteristic noise signals for mesitylene-d_{12} at 10 µM (11.5 ppm, 11 ppm, 5 ppm, −2 ppm, −2.5 ppm).

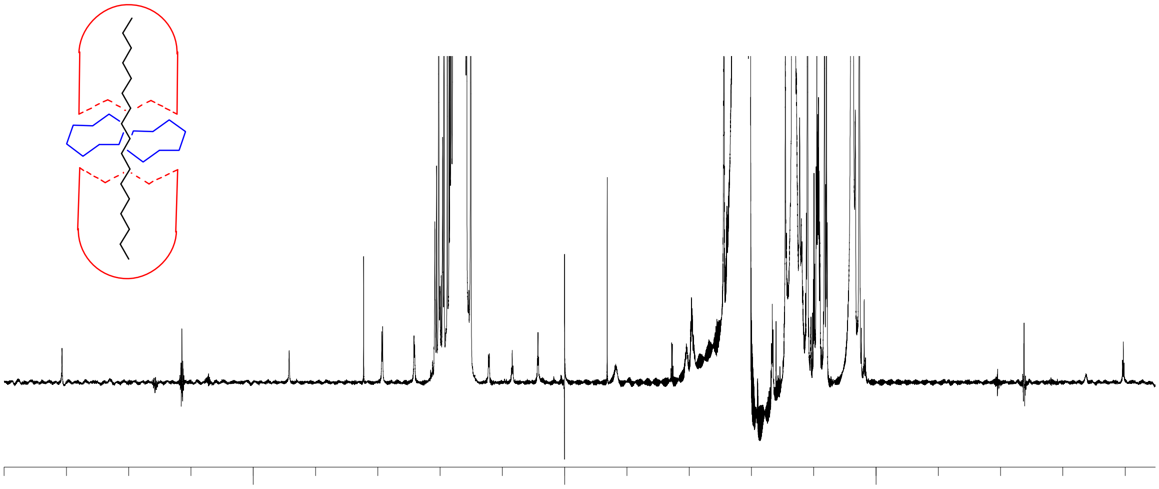

$C_{11}H_{17}$ inside **1.1** (mesitylene-d_{12}). ^{1}H NMR.

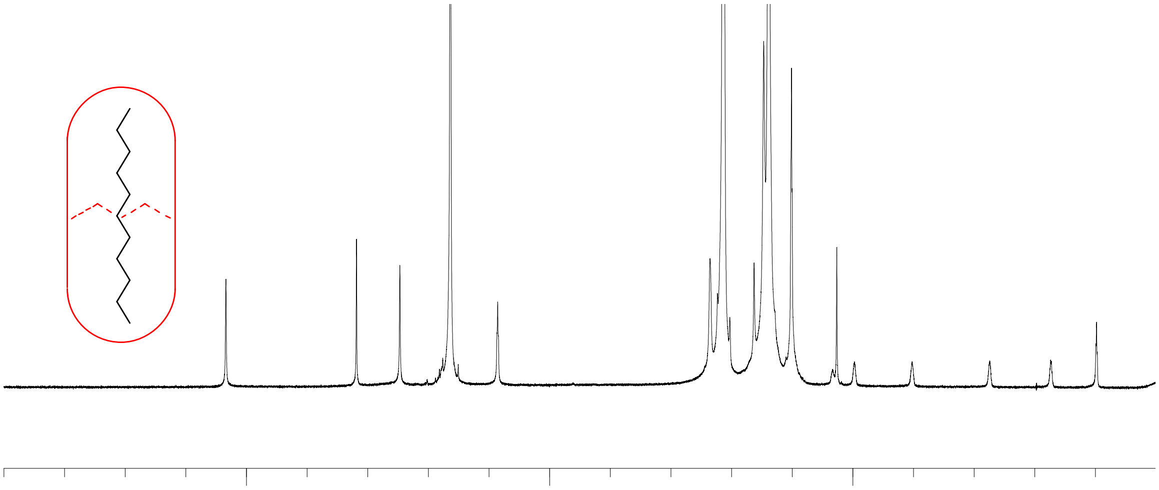

Compound **6** (mesitylene-d_{12}). ^{1}H NMR.

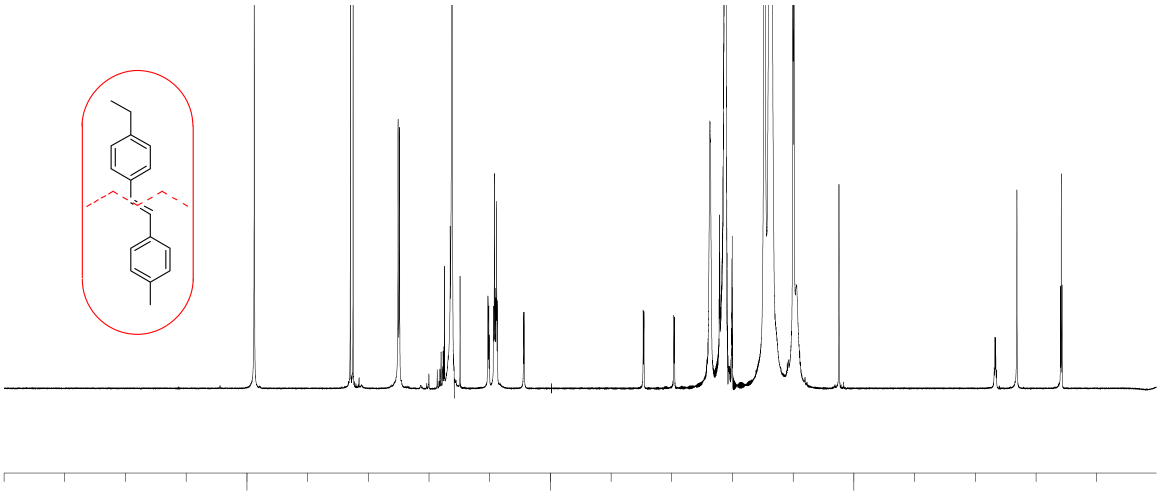

References for the Supporting Information

1. McDonald, R. N.; Campbell, T. W. Synthesis of Hydrocarbon Derivatives by the Wittig Reaction II. Diarylbutadienes and Quinquephenyls. *J. Org. Chem.* **1959,** *24,* 1969–1975. doi:10.1021/jo01094a036
2. Grasa, G. A.; Singh, R.; Stevens, E. D.; Nolan, S. P. Catalytic activity of Pd(II) and Pd(II)/DAB-R systems for the Heck arylation of olefins. *J. Organomet. Chem.* **2003,** *687,* 269–279. doi:10.1016/S0022-328X(03)00375-9
3. Barret, E. S.; Dale, T. J.; Rebek, J., Jr. Self-Assembly Dynamics of a Cylindrical Capsule Monitored by Fluorescence Resonance Energy Transfer. *J. Am. Chem. Soc.* **2007,** *129,* 8818–8824. doi:10.1021/ja071774j
